# Urine and Blood-Derived MicroRNAs in Patients with Kidney Cancer: A Review of Clinical Utility and Recent Developments

**DOI:** 10.3390/cells15161485

**Published:** 2026-08-18

**Authors:** Samuel Y. R. Chen, Serina Quach, Vladislav Nikitin, Jennifer A. Linehan, Matias A. Bustos

**Affiliations:** 1 Department of Translational and Precision Medicine, Saint John’s Cancer Institute (SJCI), Providence Saint John’s Health Center (SJHC), Santa Monica, CA 90404, USA; chen.yiren@mayo.edu (S.Y.R.C.); serina.quach@providence.org (S.Q.); vladislav.nikitin@providence.org (V.N.); 2 Department of Urology and Urologic Oncology, Saint John’s Cancer Institute (SJCI), Providence Saint John’s Health Center (SJHC), Santa Monica, CA 90404, USA; jennifer.linehan@providence.org

**Keywords:** cfmiRs, microRNA, extracellular vesicles, diagnostic, prognostic, blood, renal cell carcinoma, liquid biopsy

## Abstract

Renal cell carcinoma (RCC) is frequently detected incidentally, and no widely adopted noninvasive biomarkers are available for RCC diagnosis or prognosis. In this regard, cell-free microRNAs (cfmiRs) have emerged as promising candidates due to their stability in biological fluids. In this narrative review, we summarize translational studies published from 2010 to 2025 that evaluated serum, plasma, or urinary cfmiRs for RCC diagnosis, prognosis, recurrence surveillance, or treatment-response monitoring. Forty-two studies met inclusion criteria, comprising 3454 patients with RCC and 2445 healthy donors. Twenty-nine studies assessed diagnostic performance, fewer evaluated prognostic applications, and none examined treatment-response monitoring. Multi-miRNA panels generally reported higher performance than single-miRNA assays. Serum was the most frequently studied biofluid in this review (*n* = 26), followed by urine (*n* = 12) and plasma (*n* = 4). Urinary biomarkers demonstrated high specificity and the practical advantage of noninvasive collection. Although limited in number, prognostic studies identified associations between cfmiRs and overall survival, recurrence-free survival, metastasis-free survival, and other clinically relevant outcomes. However, substantial heterogeneity in study design, assay methods, and reporting limited comparisons across studies. Current evidence supports continued evaluation of cfmiRs as adjunctive biomarkers alongside imaging or histopathology, but multicenter validation, standardized methods, and more robust evidence are required before clinical implementation.

## 1. Background

Renal cell carcinoma (RCC) incidence rates have stabilized, even though no early detection biomarkers exist, and this rate stabilization is attributed to an increase in incidental findings [1]. RCC is a heterogeneous disease whose three most common histological subtypes are clear cell RCC (ccRCC), papillary RCC (pRCC), and chromophobe RCC (chRCC). The incidence and aggressiveness of each of the RCC subtypes vary, with ccRCC and pRCC subtypes being the most aggressive forms. Clinically, patients with RCC are often asymptomatic and diagnosis is frequently incidental. Consequently, around 20% of RCC patients will have metastatic disease at the time of diagnosis [2].

Although percutaneous renal biopsy is often regarded as a relatively low-risk and accurate diagnostic tool, it still carries meaningful risks of procedural complications, including anemia, gross hematuria, and hematoma, and may be contraindicated in hemodynamically unstable patients and other populations [3]. Moreover, renal biopsy performs poorly as a negative predictor of RCC (negative predictive value = 63.3%), further emphasizing the need for accessible and accurate methods of assessing cancer risk prior to resection [4]. Contrary to the current standard of care—where most RCCs are detected incidentally by ultrasound/computed tomography (CT)/magnetic resonance imaging (MRI) performed for unrelated indications and indeterminate renal masses often require repeated imaging, contrast exposure, and subsequent biopsy—urine and blood assessments offer a scalable, minimally invasive way to triage risk and reduce diagnostic uncertainty. Blood-based assays (serum/plasma) complement urine-based assays by capturing systemic tumor signals and may be more sensitive for small or non-communicating lesions, enabling the earlier flagging and triaging of patients who require further definitive imaging and follow-up. Urine-based assays are particularly attractive because they sample the urinary tract directly and can provide highly specific signals that may help differentiate malignant RCC from benign renal lesions, potentially limiting unnecessary surgery or prolonged imaging surveillance. In practice, integrating urine and blood biomarkers with imaging could, with more robust evidence, allow RCC diagnosis to move from opportunistic detection to a more proactive, multi-modal screening, improving early detection, supporting decision-making for small masses, and enabling low-risk longitudinal monitoring.

Because a significant subset of patients with RCCs eventually recur, initial surgical resection is often followed by a prolonged period of active surveillance to monitor for recurrence and/or metastasis. Current American Urological Association guidelines recommend a strict schedule of imaging, ranging from every three to six months depending on tumor stage and pathological grade [5]. Although this regimen is thorough, it can be resource-intensive for facilities with limited CT and MRI availability and may also expose patients to increased radiation. Urine and blood-based assessments could supplement current guidelines by providing a less invasive and less expensive method of monitoring for recurrence and/or metastasis. Diagnostic cfmiR biomarkers could enable earlier detection by allowing for more frequent follow-up, when integrated with imaging. In addition, prognostic cfmiRs may help inform postoperative chemotherapeutic or immunotherapeutic treatment decisions.

**Figure 1:**
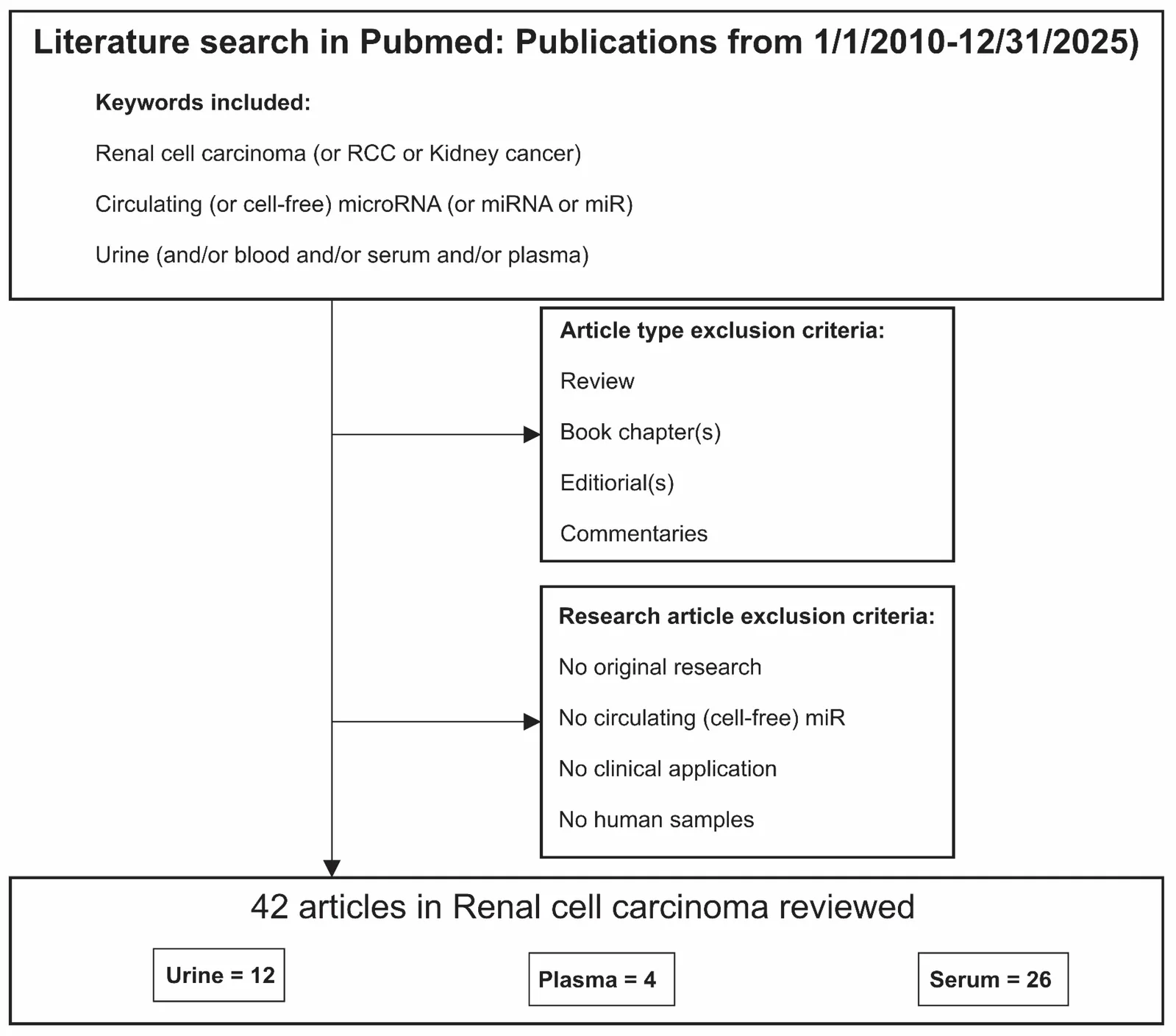
Studies included in the review. Schematic representation of the literature search and the articles included in the review.

To begin, a focused literature search was performed on English-language original publications from 2010–2025 in the PubMed database in February 2026 that explored circulating or urinary cfmiRs in RCC. Keywords included were: renal cell carcinoma (or RCC or kidney cancer), circulating (or cell-free) microRNA (or miRNA or miR), and urine (and/or blood, and/or serum, and/or plasma). After querying, original research was included, while reviews, book chapters, editorials, and commentaries were excluded. Further exclusion criteria included research with no circulating or cell-free miRNA, no clinical application, and/or no human samples. In total, 42 publications met the inclusion criteria and were included in this review (Figure 1 depicts the inclusion and exclusion criteria and the studies included). As a narrative review, the search was not prospectively registered (e.g., PROSPERO) and a formal PRISMA screening workflow was not followed.

All performance values were summarized descriptively from the estimates reported by the original articles. All averages were unweighted and intended to serve as an overview of general performance and not comprehensive pooled estimates. Further meta-analysis was not performed due to substantial study heterogeneity and incomplete reporting. Unless otherwise stated, each marker or panel estimate contributed one observation; therefore, studies that reported multiple candidate cfmiRs (e.g., as part of a panel) contributed more than one non-independent value to summary statistics.

This review summarizes the aforementioned studies with an emphasis on diagnostic and prognostic performance, inter-study heterogeneity, and recent technological developments relevant to cfmiR biomarker development.

## 2. Results

Across the studies in this review, 3454 RCC patients were compared with 2445 healthy donor (HD) controls. The vast majority of RCC patients either had no subtype reported (denoted in Table 1, Table 2 and Table 3 as RCC) or had pathology-confirmed ccRCC. Among the rarer subtypes, 3.30% (114/3454) were pathologically confirmed pRCC cases, whereas only 1.21% (42/3454) had chRCC. Although most studies used matched HDs as controls, some compared RCC patients against individuals with benign renal tumors (BRTs); 89 patients with a variety of BRTs were included as controls: 17 angiomyolipomas, 23 oncocytomas, two papillary adenomas, and 47 unspecified. Only one study compared pre-nephrectomy RCC patients with self-matched (post-nephrectomy) controls [6]. The cfmiR most represented by the included studies, by far, was miR-210/miR-210-3p, which is present in 23.8% (10/42) of all studies included in this review. The next most prevalent cfmiRs were miR-21 and miR-378, each appearing in 9.52% (4/42) of studies.

Across the 42 studies included in the review, 29 (69.0%) evaluated only the diagnostic value of cfmiRs in RCC, four (9.52%) evaluated only the prognostic value, and nine (21.4%) assessed both. Reporting practices varied across studies, but most that examined diagnostic utility reported the AUC of the ROC curve, often alongside sensitivity (SE) and specificity (SP) values for a given single cfmiR or panel. There were 110 reported AUC values in total and 70 reported SE and SP values, with many studies reporting diagnostic performance for each individual cfmiR in their relevant panel. Among these, the mean AUC was 0.746 (range = 0.545–0.970; *n* = 110; mean SE = 73.4%; mean SP = 72.7%). When comparing multi-miRNA panels against single miRNA analysis, panels achieved a mean AUC of 0.861 (range = 0.718–0.960; *n* = 18; mean SE = 85.6%; mean SP = 81.2%) compared to an average AUC of 0.723 (range = 0.545–0.970; *n* = 92; SE = 70.6%, SP = 70.7%) for single cfmiRs. Given the differences in specimen types, control selections, assay platforms, and histologic compositions across studies, cfmiR findings were subsequently examined by biofluid.

**Figure 2:**
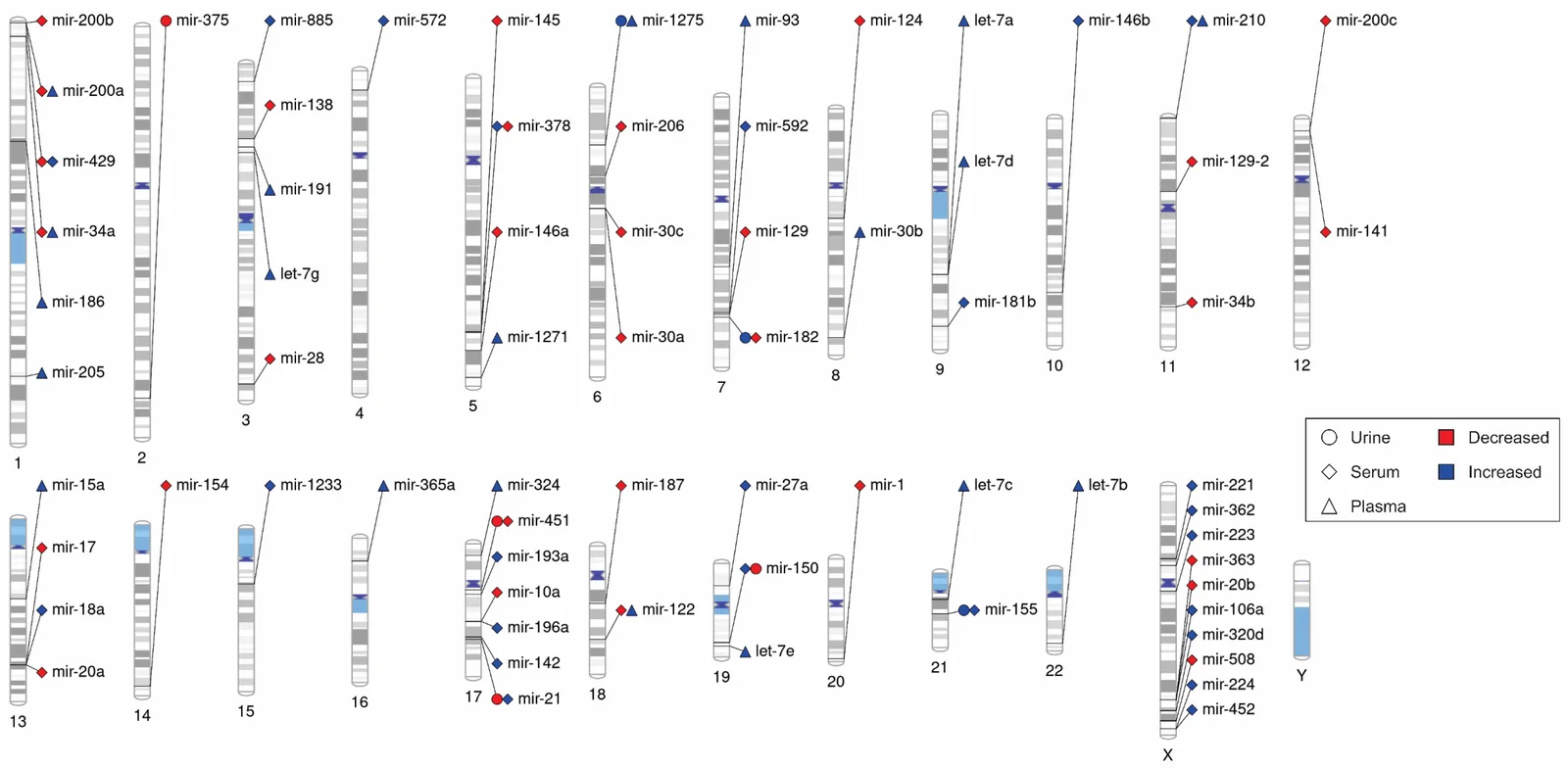
Chromosomal locations of cfmiRs included in the review. Schematic representation of the chromosomal location of the cfmiRs identified in the 42 studies included in the review.

Chromosomal mapping of the 71 unique cfmiRs showed broad distribution across all autosomes and the X chromosome without obvious chromosomal bias, although several loci contained overlapping signatures across biofluids (Figure 2, see Section 2.5).

### 2.1. Blood-Derived cfmiRs in RCC

Serum and plasma represent the two main sources of cfmiRs that reflect RCC tumor presence in blood. Recent studies have favored plasma over serum for cfmiR assays due to better reproducibility and fewer confounding factors (e.g., avoiding platelet-derived miRNAs released during clotting). However, most research into blood cfmiRs has utilized serum as the analyte, likely due to the relative simplicity in sample preparation and the commercial availability of specialized kits and protocols. Since serum and plasma may exhibit vastly different cfmiR backgrounds, blood-based studies were divided by source. Out of a total of 30 blood-based studies included, 86.7% (26/30) utilized serum and the remaining 13.3% (4/30) analyzed plasma.

### 2.2. Serum-Based cfmiRs

Serum was the first blood fraction used for cfmiR biomarker studies in RCC. Several early studies identified diagnostic serum cfmiRs, though some suffered from limited specificity and a small sample size (Table 1). Serum samples typically quantified cfmiRs with PCR-based assays (quantitative reverse transcription polymerase chain reaction (qRT-PCR) or droplet digital polymerase chain reaction (ddPCR); Table 1). In some studies, EVs were purified from serum samples [14,22]. cfmiRs in RCC patients were either compared against a defined cohort of HDs (92.3%), patients diagnosed with benign tumors (3.85%), or localized ccRCC (3.85%) as the control group. In accordance with their prevalence, serum studies included 67.2% (2324/3454) of total RCC patients, comparing them against 71.6% (1751/2445) of total HDs. One serum study [16] analyzed only pRCC subtype, which made up 58.8% (67/114) of total pRCC cases, while, notably, no serum studies included patients with chRCC subtype. Concordant with the general bias towards diagnostic studies, 17 studies (65.4%) analyzed diagnostic performance only, two studies (7.7%) analyzed prognostic performance only, and seven studies (26.9%) incorporated both in their evaluation of cfmiRs.

miR-1233 was one of the first reported RCC-associated cfmiRs. In a 2011 multi-center study [32], serum miR-1233 levels were significantly elevated in patients with RCC vs. HDs. The initial performance was modest (a sensitivity of ~77%, but a specificity of only ~38%, and an AUC of 0.59), indicating many false positives [32]. Notably, miR-1233 was also elevated in patients with BRTs in that study, possibly explaining the low specificity for RCC [32]. This illustrates that some cfmiRs lack cancer specificity and underscores the need for better markers or panels in serum.

Later research identified serological miR-210 as a consistent top candidate in RCC tissue studies. A 2014 study in patients with early stage ccRCC reported that serum miR-210 alone achieved a 65% sensitivity and 83% specificity (AUC ~0.77, false positive rate = 17%) in distinguishing RCC from HDs [28]. A 2017 meta-analysis (seven studies, *n* ≈ 570 RCC cases) confirmed circulating miR-210’s diagnostic value with a pooled 74% sensitivity, 76% specificity and an AUC of ~0.81 [47]. These results suggest serum miR-210 is a moderately accurate biomarker for RCC but with significant false negative and false positive rates (26% and 24%, respectively).

**Figure 3:**
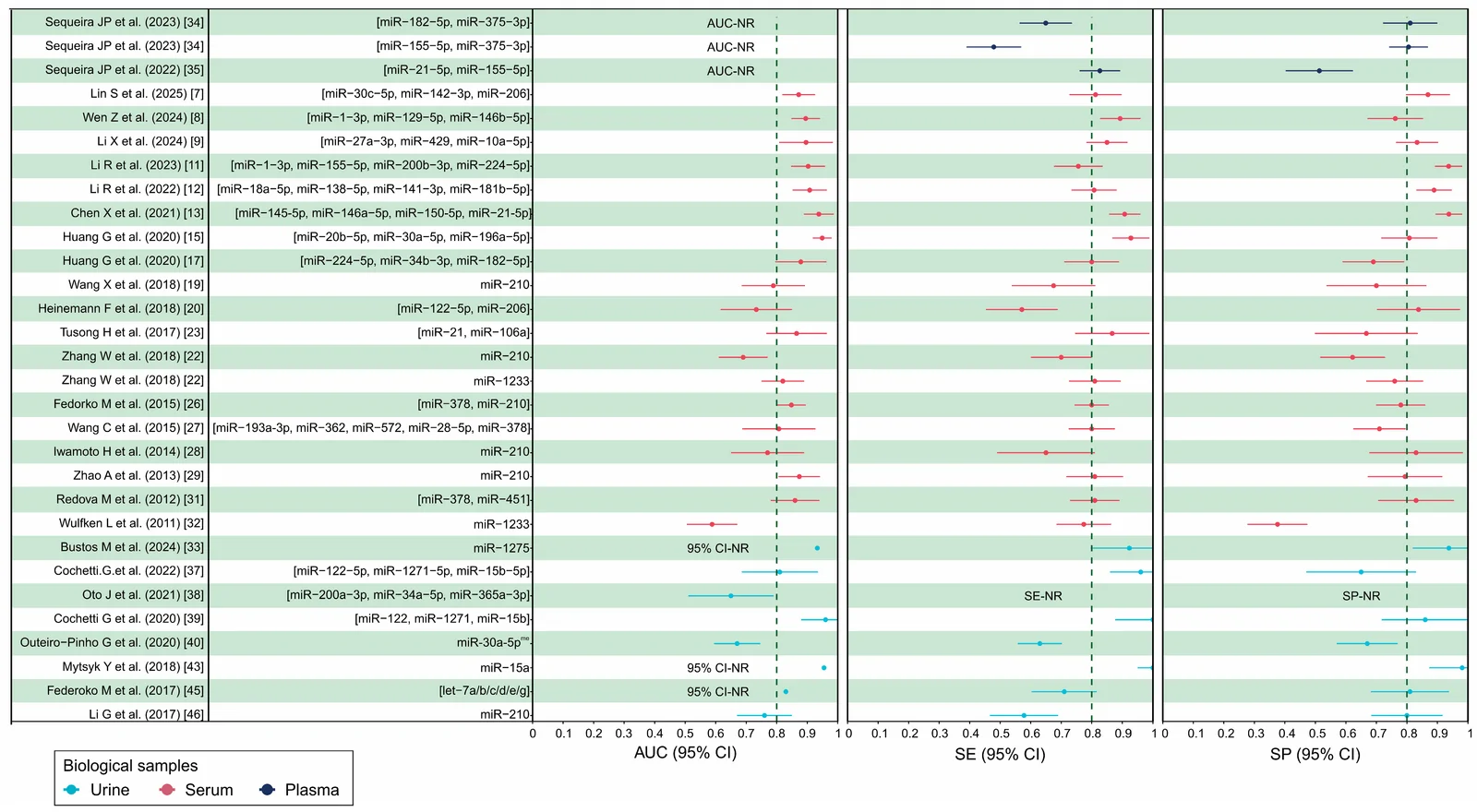
cfmiR panels and their diagnostic performance depending on the biofluid analyzed. Descriptive forest plots showing the diagnostic performance of cfmiR panels selected from the 42 studies included. Dots indicate the reported AUC, and the lines demarcate the 95% confidence interval. If a 95% confidence interval was not reported the reported AUC is represented by a singular dot. Studies that did not evaluate a panel are represented by a single reported cfmiR signature. The plotted entries are intended for descriptive cross-study comparisons and are not a pooled meta-analysis or statistical ranking. AUC = area under the curve; CI = confidence interval; NR = not reported.

To improve single-marker performance, researchers have largely turned towards serum cfmiR panels [7,8,9,10,11,14,16,17,18,19,20,21,22,23,24,25,27,28,29,30,31,48,49,50,51,52,53,54,55,56,57,58,59,60,61,62,63,64,65,66,67,68,69,70,71,72,73]. For example, a 2015 study identified a panel of five serum cfmiRs (miR-193a-3p, miR-362, miR-572, miR-28-5p, miR-378a-5p) for early RCC detection. In a validation cohort, this 5-miRNA panel achieved approximately 80% sensitivity and 71% specificity (AUC ~0.80) for detecting stage I RCC versus controls [27]. Similarly, panels combining miR-210 with other markers have shown improved diagnostic accuracy over any single cfmiR alone [13,35]. These findings align with other cancer studies: panels of multiple cfmiRs outperform individual markers in both sensitivity and specificity. Serum cfmiR tests have demonstrated proof-of-concept for RCC detection, but earlier single-cfmiR tests (e.g., miR-1233) were not sufficiently specific for clinical use. Newer multi-cfmiR panels in serum show a more balanced diagnostic profile (sensitivities of ~70–80% with acceptable specificities around 70–80% in early disease characterized by clinical stage and tumor size). In aggregate, serum studies demonstrated modest diagnostic performance, with an average AUC of 0.752 (range = 0.545–0.970; *n* = 79; mean SE = 75.5%, mean SP = 72.1% across 49 reported values). Serum multi-miRNA panels, a common research direction, accounted for 69.5% (16/23) of the total panels included in this review. These panels demonstrated a mean AUC of 0.864 (range = 0.718–0.938; *n* = 14; mean SE = 83.3%; mean SP = 82.2%), with higher reported performance in multiple diagnostic criteria when compared to single cfmiR studies (mean AUC = 0.728, range = 0.545–0.970; *n* = 65; SE = 73.2%, SP = 69.2%) (Figure 3).

Beyond diagnostics, serum cfmiRs have also demonstrated emerging prognostic utility in RCC, although the relevant body of literature remains smaller and more heterogeneous than diagnostic literature. Several recent studies have linked altered serum cfmiR expression with overall survival (OS), progression-free survival (PFS), cancer-specific survival (CSS), disease-free survival (DFS), recurrence, Fuhrman grade, and advanced disease status (Table 1). Multiple recent qRT-PCR studies in ccRCC identified serum cfmiR signatures associated with OS. Each study initially explored a different set of three or four cfmiRs in the following panels: [miR-1-3p, miR-129-5p, miR-146b-5p], [miR-27a-3p, miR-429, miR-10a-5p], [miR-1-3p, miR-155-5p, miR-200b-3p, miR-224-5p] and others [8,9,11]. Importantly, only individual cfmiRs were evaluated for prognostic value. The same studies also showed diagnostic discrimination, suggesting that some serum cfmiR signatures may have dual clinical value, consistent with cfmiR dysregulation associated with RCC, though this remains to be demonstrated in larger cohorts.

**Figure 4:**
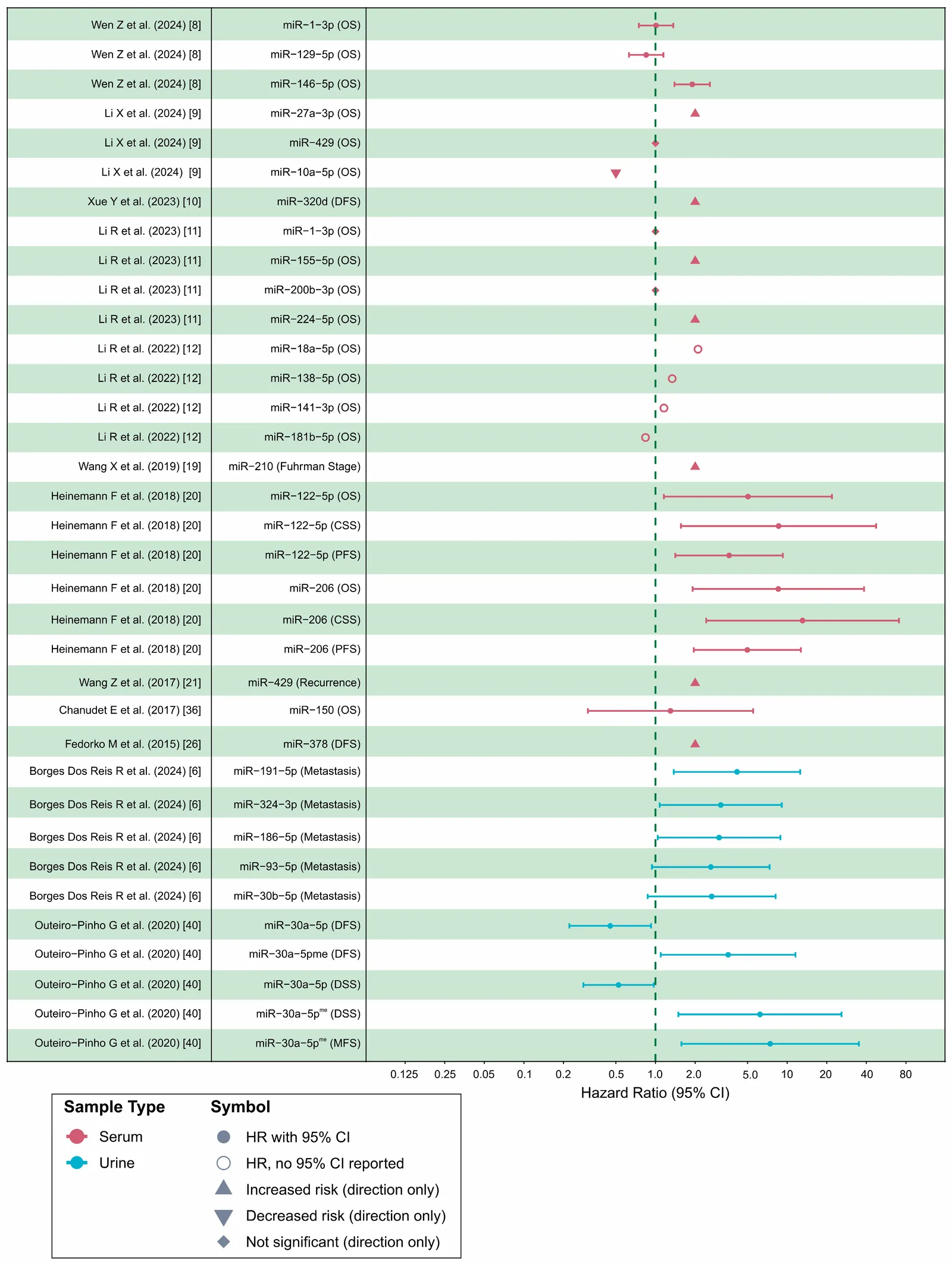
cfmiRs with prognostic utility. Descriptive forest plot showing reported hazard ratios for prognostic cfmiRs from the studies included in this review. When no numerical HR was reported, triangles are placed at arbitrary reference positions (0.5/1.0/2.0) solely to indicate direction and should not be interpreted quantitatively. Open circles indicate HR values reported without a 95% CI or standard error. HR = hazard ratio; CI = confidence interval; NR = not reported.

Several individual serum cfmiRs were likewise associated with adverse clinicopathologic features in their individual studies. Serum miR-320d was increased in advanced versus localized ccRCC [10], supporting an association with increasing tumor burden, while serum miR-210 was reported to have a strong positive correlation with Fuhrman grade in the exosome fraction [19]. A study also assessed miR-122-5p and miR-206’s association with a variety of prognostic measures, including OS, CSS, and PFS (Figure 4) [20]. In contrast to diagnostic measures, the reporting of prognostic utility was especially heterogeneous. Studies focused on a variety of clinicopathologic features and reported varying amounts of statistics from simple *p*-values to hazard ratios (HRs) with confidence intervals (CIs). Where reported, the HRs trend in the expected direction, though confidence intervals are often large and variable, even when assessing the same cfmiR (Figure 4).

### 2.3. Plasma-Derived cfmiRs

Plasma studies represented the smallest proportion of the literature included, comprising only four studies with 10.2% (355/3454) of RCC patients, compared against 12.7% (311/2445) of HDs. In contrast to the small number of studies, the plasma cohort contained the most histologically diverse patient population of the three biofluids, with one study [35] including three separate subtypes: pathologically confirmed ccRCC (87), pRCC (15), and chRCC (22) patients and accounting for 13.2% (15/114) of all pRCC cases and 52.4% (22/42) of all chRCC cases across the review. Like serum studies and overall trends in RCC cfmiR research, three plasma studies (75.0%) analyzed diagnostic performance only and one (25.0%) incorporated both diagnostic and prognostic evaluation. Of note, no plasma study assessed prognostic performance exclusively. There was considerable heterogeneity in methodology among plasma studies, with two studies employing ddPCR [34,35], one using NGS [33], and one relying on qRT-PCR [36].

In general, individual plasma cfmiR biomarkers for RCC have shown variable results. For instance, miR-21-5p, a commonly elevated cfmiR that is implicated in many cancers, is found at higher levels in RCC patients’ plasma, but results have been mixed on its standalone accuracy. These inconsistencies, just as with serum studies, have prompted a shift toward multi-cfmiR panels.

A 2022 study by Sequeira et al. developed a 4-miRNA panel (“LiKidMiRs”) for plasma-based RCC detection [35]. A two-microRNA combination (miR-21-5p + miR-155-5p) could detect ~83% of RCC cases with 72% overall accuracy. Notably, this plasma cfmiR pair correctly identified 89% of early stage RCC cases, indicating clinical value for early detection. While specificity was more modest (~52% when both markers were used to distinguish clear-cell RCC from other subtypes), the high sensitivity suggests such panels could serve as effective rule-out tests for initial RCC screening [35]. One potential advantage of plasma is stability and consistency; Sequeira et al. found that plasma cfmiR profiling was successful in RCC patients [35] and reported high-sensitivity plasma panels (e.g., miR-21 + miR-155) which achieved an 82.7% SE.

Overall, plasma studies demonstrated the most modest diagnostic performance of the three biofluids, with an average AUC of 0.668 (range = 0.581–0.801; *n* = 10; mean SE = 56.6%; mean SP = 71.1%). The notably lower average SE relative to serum and urine likely reflects the inclusion of several Sequeira et al. cfmiR panels [34,35] that prioritized specificity over sensitivity in their experimental design. Plasma panels accounted for a similarly modest 13.0% (3/23) of panels in this review, and 75% (3/4) reported at least one panel AUC. However, two of the panel AUCs were reported without corresponding SE or SP values, limiting conclusions on diagnostic performance.

Due to the low overall number of plasma studies, only one study (out of four) examined the prognostic utility of plasma cfmiRs in RCC [36]. The study found that low miR-150 levels were significantly associated with shorter PFS (HR: 1.3, 95% CI [1.0–1.8]). Much remains to be determined of the prognostic value of other cfmiRs in plasma.

### 2.4. Urine cfmiRs: High Specificity and Noninvasive Sampling

Several urinary cfmiRs have shown diagnostic potential as shown in a recent systematic review by Cochetti et al. (Table 3). Across the 12 urine cfmiR studies summarized in Table 3 and Figure 3, diagnostic performance ranges from modest single-analyte discrimination (AUC ~0.65–0.76 for several individual markers) to higher reported performance for selected single markers and multi-miRNA panels (AUC ~0.81–0.96) [33,37,38,39,40,43,46,74]. These studies comprised 22.4% (775/3454) of total RCC patients, compared against 15.7% (383/2445) of total HDs. Unlike serum and plasma studies, urine studies more frequently included BRT controls (*n* = 27), with one study [43] comparing RCC patients against a combined control group of HDs and many BRTs, including oncocytomas (eight), papillary adenomas (two), and angiomyolipomas (four), and another [38] including angiomyolipomas (13) in their control group. Notably, one urine study [6] employed a self-matched design, comparing pre-nephrectomy and post-nephrectomy urine samples rather than matched HDs. Regarding subtypes, urine studies included 32/114 (28.1%) of all pRCC cases and 20/42 (47.6%) of all chRCC cases across the review. Two urine studies (16.7%) evaluated prognostic performance exclusively, the highest proportion out of the three biofluids, while nine (75.0%) assessed diagnostic performance only, with only one study (8.3%) incorporating both. Regarding the detection of cfmiRs, urine studies were the most methodologically homogeneous of the three biofluids, with 11 of 12 studies employing qRT-PCR [6,37,39,40,41,42,43,44,45,46] and only one utilizing NGS [33]. However, urine studies were more heterogeneous in what fraction of urine was analyzed (whole, supernatant, or precipitate), with one study isolating extracellular vesicles (EVs) for cfmiR analysis [70].

Urinary miR-15a was dramatically elevated in RCC patients and dropped by ~99% post-nephrectomy, indicating a tumor origin. Elevated levels of miR-15a distinguished RCC from BRTs with 100% sensitivity and 98% specificity (AUC ~0.96) in the initial cohort with 22 ccRCC patients (Table 3 and Figure 3). miR-210 has likewise been frequently studied in urine with a 2017 study reporting that urinary miR-210 could detect clear-cell RCC with an AUC of ~0.76 (SE = 58%, SP = 80%) [46]. While moderate in accuracy, miR-210 levels consistently decrease after surgery, supporting its potential utility for detecting tumor-associated changes and post-nephrectomy surveillance (Table 3 and Figure 3). Research has also demonstrated elevated urinary miR-30c-5p in RCC patients with a reported AUC of ~0.82 (SE = 68.6%, SP = 100%) in differentiating RCC from controls [42] (Table 3 and Figure 3).

In accordance with serum panels, multiple urinary cfmiR panels often improve diagnostic performance. For example, a “7p-urinary” miRNA score (combining miR-122-5p, miR-1271-5p, and miR-15b-5p and using various internal controls including miR-16-5p, Cel-miR-39-3p, and miRTC) achieved an initial AUC of 0.96, and an AUC of 0.81 upon independent validation, outperforming single markers in accuracy [37]. Likewise, other studies have noted that let-7 family miRNAs and miR-210 are frequently upregulated in RCC urine and warrant inclusion in multi-marker panels. These panels demonstrate that urinary cfmiR signatures can reach 0.80–0.96 AUC with high SE/SP in small studies, although consistency across different cohorts remains uncertain [45]. When comparing signatures across studies in Table 3, only a limited number of cfmiRs recur, underscoring heterogeneity in discovery methods, data normalization and methodology, and variability across different cohorts of patients [33,37,38,39,40,43,46,74]. The only consistent overlap is miR-210/miR-210-3p [41,46].

Urine studies demonstrated a high diagnostic performance with an average AUC of 0.759 (range = 0.590–0.960; *n* = 21; mean SE = 75.7%; mean SP = 75.5% across 13 reported values). Single cfmiR performance in urine was higher than other biofluids (mean AUC = 0.742; range = 0.590–0.955; *n* = 18), but this increase was driven in part by the high-performing miR-15a (AUC = 0.955), which did have a notably low sample size [43]. Urine panels demonstrated slightly better performance, achieving a mean AUC of 0.867 (range = 0.810–0.960; *n* = 3). However, this figure is heavily skewed by the Cochetti et al. 3-cfmiR panel (miR-122/miR-1271/miR-15b), which reported an AUC of 0.96 in the discovery cohort and may reflect a certain amount of overfitting [39]. Urine panels accounted for only 17.4% (4/23) of total panel-reporting studies in this review, and of these, two panel AUCs were reported without corresponding SE or SP values, limiting comparison to biofluids, like serum, with more reported values. While urine studies were fewer in number and smaller than serum studies, their reported diagnostic performance was encouraging across several metrics, supporting further evaluation of urine as a promising biofluid for cfmiR-based RCC detection.

Studies have also analyzed the utility of urinary cfmiRs in the prognosis of RCC. Early studies focused on miR-30a-5p as a marker for metastasis and disease progression. Specifically, they found that decreased urinary miR-30a-5p levels and increased miR-30a-5p promoter methylation were significantly associated with shorter MFS and DSS [40]. More recent studies have evaluated other cfmiRs, with miR-191-5p, miR-324-3p, and miR-186-5p all independently demonstrating significant association with metastasis. Although these findings support prognostic relevance, HRs and CIs (which ranged from 1.5–40), demonstrated high individual variability that may make prognostic conclusions challenging in clinical environments [6] (Table 3 and Figure 4).

### 2.5. Chromosomal Locations and Overlapping Signatures

The 71 unique cfmiRs identified across 42 studies were mapped to their respective chromosomal loci to assess whether RCC-associated cfmiRs are clustered by genomic locus or biofluid (Figure 2). miRNAs appear across all autosomes and the X chromosome, with no apparent chromosomal bias in the concentration of clinically relevant miRNAs. Within certain chromosomes, such as chromosomes 1 and X, dysregulated miRNAs appear to cluster near the ends of chromosomes. On the other hand, certain chromosomes (e.g., chromosome 17) do not appear to exhibit such bias, and miRNAs are mapped throughout. Several chromosomal regions contained miRNAs identified across more than one biofluid, with one example being chromosome 17, which harbors both miR-21 and miR-451, both identified in plasma and serum samples. Ten unique miRNAs were mapped to chromosome X, all of which were analyzed only in serum samples, which may suggest some bias in miRNA selection by biofluid (as newer serum studies may look towards previously unanalyzed miRNAs due to the prevalence of serum cfmiR research compared to research in urine or plasma). Interestingly, the directionality of expression (increased vs. decreased in RCC relative to HDs) was not uniformly conserved even within the same chromosomal region, with some loci harboring both upregulated and downregulated cfmiRs depending on the study, biofluid analyzed, and methodology utilized. Chromosomal mapping was exploratory and intended to illustrate the genomic distribution of the reported cfmiRs as well as overlapping locations across biofluids. Whether enrichment at particular chromosomal loci contributes to their clinical performance in RCC diagnosis and prognosis was not established by the included studies.

cfmiR expression profiles in RCC often vary substantially across studies due to differences in patient cohorts, clinical variables, underlying biology, and the specific biofluid analyzed. For example, miR-122 was reported to be downregulated in the serum of patients with the ccRCC subtype [20], whereas another study documented upregulation of miR-122 in urine samples [37,39]. Similarly, miR-200a shows conflicting findings: Oto et al. (2021) reported elevated pre-operative urinary levels in ccRCC patients, while Wen et al. and Li et al. observed downregulation of miR-200a in serum samples [8,38]. These discrepancies (particularly for miR-200a) may reflect methodological differences in data normalization, including the choice of endogenous controls (e.g., use of miR-20a-5p as a urinary reference cfmiR but use of a different reference in serum samples).

Similar inconsistencies are observed even among blood-derived analytes, particularly when comparing plasma and serum. Sequeira et al. [34] reported increased miR-182 levels in plasma, whereas Huang et al. [17] found significantly decreased miR-182 levels in serum from ccRCC patients relative to HDs. Likewise, Sequeira JP et al. [34] was the only study to observe decreased plasma miR-21-5p in RCC, while two other studies reported increased miR-21-5p in serum from ccRCC patients [13,16]. These discordant findings may be driven in part by methodological differences in cfmiR quantification, as Sequeira et al. [34] used ddPCR whereas the serum-based studies employed qRT-PCR. Notably, conflicting directionality has also been reported within the same biofluid: multiple early studies [26,31] reported increased serum miR-378a-5p, while Wang C et al. alone reported decreased levels [27]. Altogether, these inconsistencies signal caution in generalizing single-center or low sample size studies to broader populations. These differences also support the growing body of research focusing on panels of three or more cfmiRs to increase diagnostic accuracy while working with heterogeneous datasets.

### 2.6. Clinical Applications in Other Renal Pathologies

**Figure 5:**
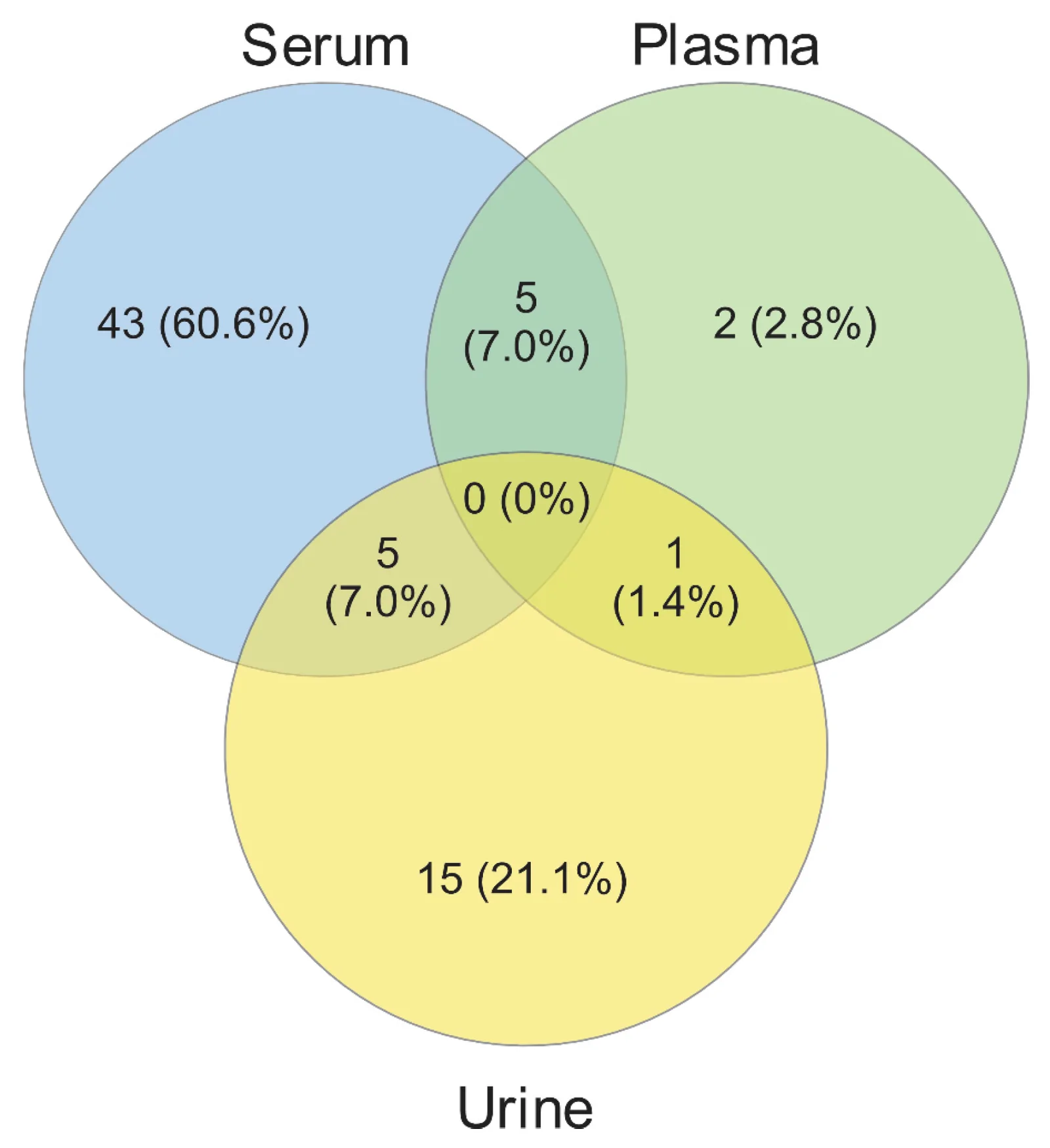
Overlapping cfmiRs with clinical utility in different biofluids. Venn diagram showing the overlapping cfmiRs identified in serum, plasma, and urine samples from patients with RCC.

Across all of the studies included in the review, very few cfmiRs demonstrated overlap across different biofluids, underscoring the heterogeneity in cfmiR detection (Figure 5). Interestingly, no cfmiR was commonly detected across all three biofluids (serum, plasma, and urine). This lack of a tripartite overlap may have been due in part to the small number of studies assessing plasma cfmiRs relative to urine or serum cfmiRs in RCC cohorts. Across two different biofluids, more overlaps are present, with five cfmiRs overlapping between plasma and serum (miR-155-5p, miR-182-5p, miR-21-5p, miR-150-5p, and miR-451), another five different cfmiRs overlapping between serum and urine (miR-122-5p, miR-200a-3p, miR-210-3p, miR-30a-5p, and miR-34a-5p), and just one cfmiR, miR-1275, overlapping between plasma and urine (Figure 5).

A similar pattern of overlap was observed when comparing cfmiRs in RCC with those identified in other kidney diseases (Table 4) [75]. cfmiRs that were shared between both RCC and non-malignant kidney disease were miR-223, miR-155, miR-21, miR-15a, miR-124, let-7a, and miR-30b. In chronic kidney disease (CKD), miR-223 was consistently reported as decreased, whereas in RCC it was reported as increased (Table 4) [7,75]. miR-155 was studied across CKD, diabetic kidney disease (DKD), and RCC, with variable findings noted depending on disease and sample measured. miR-21 was also identified across CKD, DKD, and RCC, with most studies reporting increased levels, with one plasma study reporting decreased levels in RCC [35]. miR-15a overlapped between DKD and RCC and was reported as increased in both conditions, while miR-124 was studied in DKD and RCC but showed mixed expression in DKD and decreased expression in RCC (Table 4) [75].

Additional overlap was observed for let-7a and miR-30b. let-7a was reported in DKD with decreased levels in plasma, urine, and serum, whereas RCC studies reported increased expression. miR-30b was also identified in both DKD and RCC, with DKD studies reporting variable findings across different biofluids and RCC studies reporting increased urine levels [6]. These results show that several cfmiRs were studied across malignant and non-malignant kidney diseases, but the direction of dysregulation was not uniform. Moreover, many of the cfmiRs identified in this review were not studied in either DKD or CKD, and a similar number were studied exclusively in non-malignant kidney diseases, highlighting the importance of disease context when interpreting cfmiR signatures.

### 2.7. Emerging Platforms for cfmiR Analysis

qPCR remains a gold standard for cfmiR analysis, but recent advancements have led to versions with enhanced precision and specificity. For example, PROMER technology utilizes DNA-RNA-DNA hybrids along with RNase H2, enabling the detection of only perfect matches. This method is sensitive enough to measure 1–10 copies and is highly suitable for liquid biopsies. SPLICER-qPCR, on the other hand, employs SplintR ligase and RNase H to differentiate mature cfmiRs more accurately from their precursors as well as closely related family members. Another development, multiplex digital PCR, facilitates the absolute quantification of cfmiRs while detecting multiple targets within a single reaction [94]. Additionally, LNA-enhanced systems use locked nucleic acid primers, delivering highly specific detection, particularly in samples with low RNA abundance [95]. While novel, these advancements are clinically practical due to the relative similarity of their workflows when compared to traditional liquid biopsy technologies. Their clinical utility remains to be demonstrated as increases in precision and sensitivity are weighed against the increased cost and complexity of these PCR improvements.

Emerging nanotechnologies and biosensors are also being recently developed for cfmiR analysis. Nanopore sensing detects individual cfmiR molecules as they pass through nanopores, which allows for multiplexed analysis without extensive sample preparation. CRISPR-Cas systems harness enzymes like Cas12a and Cas13a for both recognition and signal generation of target cfmiRs, often eliminating the need for reverse transcription steps. Plasmonic biosensors, including SPR and SERS technologies, provide label-free or ultrasensitive cfmiR detection by measuring changes in optical properties on metal surfaces [95]. Due to their novelty and divergence from traditional methods, nanotechnologies and biosensors have largely been demonstrated as proof-of-concept in recent literature. The lack of standardized protocols and the variability in nanotechnology fabrication currently limit the clinical feasibility of these advances. Improvements in machine learning and artificial intelligence have allowed their use on large datasets to facilitate the discovery of high-accuracy cfmiR panels for the diagnosis of a variety of malignancies, including RCC [96]. Within RCC, recent artificial intelligence advances have further allowed for the discovery of large (73-miRNA) panels to differentiate between ccRCC, pRCC, and chRCC with over 90% accuracy [97]. These recent advancements may allow future cfmiR panels to be elucidated from large, independent, and multi-center datasets before validation within a smaller cohort, in contrast to the single-cohort-only approach used by many studies included in this review.

For high-throughput and spatial applications, methods such as NanoString nCounter offer barcode-based, direct counting of cfmiRs, facilitating fast biomarker validation. Spatial transcriptomic analysis enables the visualization of how cfmiR expression patterns vary regionally within tissues. Finally, point-of-care testing has become increasingly feasible with portable sensors that connect to personal devices, making rapid on-site quantification of cfmiRs accessible in clinical settings.

## 3. Discussion

This narrative review of 42 studies has highlighted both the promise and fragmentation in the field of cfmiR research in RCC. Many studies explored cfmiRs with biological relevance and demonstrated unique cfmiR signatures that performed well diagnostically and prognostically. Yet, despite over 15 years of research, aggregate measures of diagnostic performance for single cfmiRs and panels, across each of the three biofluids analyzed, do not consistently reach performance levels that would support use as standalone clinical diagnostic tests.

Serum and urine cfmiR performance was comparable, with urine studies demonstrating a slightly higher diagnostic utility; however, no meta-analytic comparison was performed, and differences in study design, cohort composition, and assay methodology preclude firm conclusions regarding the superiority of any one biofluid. In practice, either a serum or urine-based cfmiR test could become an aid in early RCC screening for high-risk patients or in evaluating indeterminate kidney masses before resorting to percutaneous biopsy. Plasma studies typically had lower reported AUCs than either of the previously discussed biofluids, which may be due to relatively lower sample sizes and novel workflows. Across all biofluids, panels averaged higher performance metrics than single cfmiRs, suggesting that the biological heterogeneity of RCC is more effectively captured by multiple biomarkers in conjunction. However, there were individual cfmiRs that were reported to have higher performance than select panels, suggesting that careful inclusion criteria and normalization techniques should be employed in designing a panel. Although these statistics provide encouraging evidence for cfmiR panels, the apparent performance of cfmiR signatures must be carefully interpreted in the context of substantial methodological heterogeneity. The calculated means do not account for non-independent measures, data normalization, or assay platform limitations. Many investigations used small, single-center, retrospective cohorts, comparing patients with pathologically confirmed RCC against healthy volunteers. Such designs may exaggerate diagnostic capabilities relative to the intended clinical setting, relevant comparisons include benign renal masses, inflammatory or chronic kidney disease, and other possible neoplasms. Cohorts were also disproportionally dominated by ccRCC, which restricts the generalizability of cfmiR markers to pRCC and chRCC. In addition, many signatures were derived and evaluated within the same or closely related cohorts, with limited independent external validation.

Of the cfmiRs included in studies of this review, a few recurred, including miR-210, miR-21, miR-378, miR-155, and miR-1233. Their representation in the literature is unsurprising given they have been variously implicated in endogenous tumor suppression, apoptosis, cell-cycle regulation, and angiogenesis. miR-210, despite being the miRNA represented by the most studies in this review, has somewhat uncertain implications in RCC pathology. Previous studies have implicated miR-210 in centrosome amplification, leading to chromosomal instability, and also to a possible downregulation of E2F [98]. Conversely, miR-210 may also have a tumor suppressive effect through downregulation of Twist-related protein 1 (TWIST1), a protein involved in metastasis and evading apoptosis [99]. Increased miR-21 levels have also been demonstrated to disrupt the cell cycle through the PTEN/Akt pathway, ultimately leading to upregulation of NFκB and cyclin D1 [100]. miR-155 has been shown to interact with lncRNA TCL6. High levels of miR-155 upregulate Src/Akt signaling in RCC cells, thereby promoting epithelial-to-mesenchymal transition [101]. Other miRNAs have less established pathways. Some evidence has demonstrated that miR-1233 suppression of acetyl-coenzyme A acetyltransferase 2 (ACAT2) may contribute to ccRCC progression, though exact mechanisms remain unclear [102]. Similarly, miR-378-5p has been shown to suppress RCC proliferation and invasion, without a definite mechanistic target [103]. Taken together, the recurrent detection of these cfmiRs may reflect their involvement in RCC pathology, although their multifaceted and sometimes contradictory actions emphasize that their use in diagnostic and prognostic panels does not establish clinical utility as drug targets.

The limited overlap of studied cfmiRs and significant differences within RCC samples may reflect both biological and technical differences between serum, plasma, and urine samples. These biofluids may indeed capture vastly different RCC-correlated changes. Compared to blood-derived samples, which may detect systemic responses to malignancy (i.e., inflammation, immune activation), urine samples may capture cfmiRs related to more local and RCC-specific processes due to their proximity to the kidney. This is supported by the significant difference in the expression profile of the same cfmiR in RCC versus DKD or CKD.

**Figure 6:**
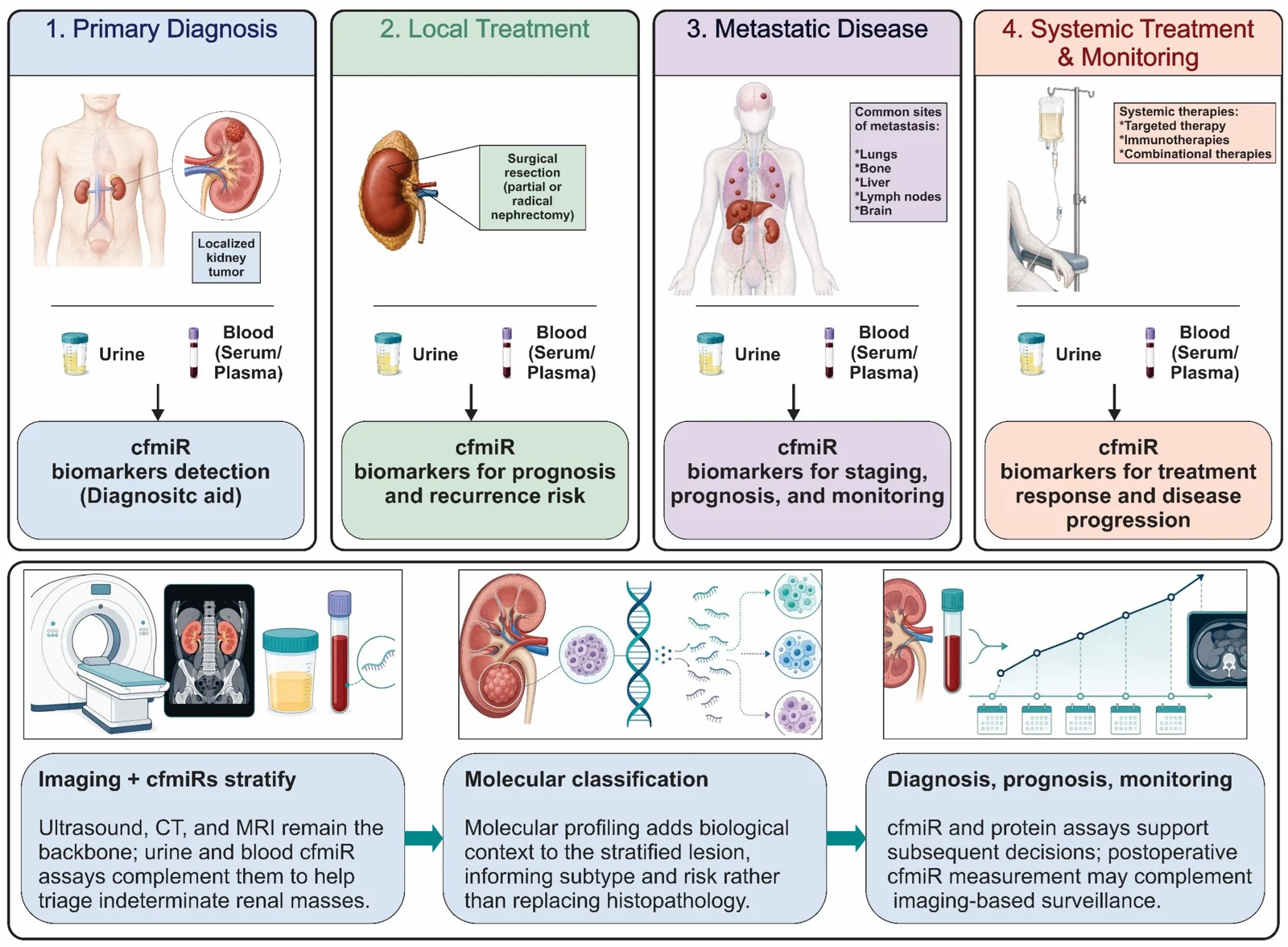
Schematic representation of potential clinical applications of cfmiRs and combinational strategies for patient care. All applications shown are conceptual and investigational; treatment-response monitoring remains unvalidated in the reviewed literature.

The performance of single cfmiR signatures also reveals potential for multimodal integration. Rather than being standalone replacements for current diagnostic tools, cfmiRs may be most useful when combined with imaging, digital pathology, and other circulating analytes (Figure 6). cfmiR quantification may complement imaging as an entry-point for renal mass diagnosis. Post-surgery, prognostically relevant cfmiRs could help stratify risk alongside quickly developing tools in digital pathology. A particularly relevant avenue of research is the development of protein biomarkers for RCC, including soluble MAdCAM-1, KIM-1, NGAL, and CRP [104,105]. In the future, a combined panel of cfmiRs and protein biomarkers may provide a more stable and clinically adaptable model than any single type of biomarker alone.

A key challenge for future RCC diagnosis is that RCC is no longer adequately represented by histologic labels alone. The 2022 WHO classification emphasized that renal tumors comprise molecularly diverse groups with distinct morphological features and clinical trajectories, which has important implications for biomarker development [106]. Many cfmiR studies were performed in cohorts dominated by ccRCC or non-subtyped RCC, which may obscure specific signals, limiting generalizability to rarer cases. As such, future cfmiR development may be more impactful when directed towards subtype-aware stratification and subsequent prognosis than for diagnosis alone. Linking circulating cfmiR patterns to a specific molecular subtype could become increasingly valuable as RCC classification becomes genomically anchored rather than histologically defined.

Another notable gap in the field is the relative lack of work in metastatic disease and active surveillance. These differences may arise due to the difficulty in analyzing multi-dimensional prognostic data versus the relative ease in determining diagnostic relevance. Although several cfmiRs have been associated with survival, recurrence, and metastasis, most studies used measurements from a single timepoint and did not assess prognostic value beyond established models. These findings therefore indicate preliminary prognostic relevance rather than validated recurrence prediction. Evidence for longitudinal surveillance and treatment monitoring is even more limited. Few studies compared pre- and post-nephrectomy measurements, and none evaluated serial cfmiRs during systemic therapy. It remains unknown whether cfmiR changes detect residual disease, precede radiographic recurrence, or predict treatment response, including response to immune-checkpoint inhibitors. These applications require prospective studies with standardized sampling, prespecified thresholds, and comparison with existing clinical tools.

Many inconsistencies were also noted across different biofluids and even within the same one. Although the reason for these may be speculated upon, it cannot be determined whether these differences are reflective of biological, methodological, or analytical differences without validation in larger samples with standardized workflows. In blood-based studies, for example, even minor hemolysis can release abundant erythrocyte-derived cfmiRs and obscure tumor-associated signals which may be less abundant [107]. Although some studies explicitly controlled this effect, other research was far more opaque about standardization. Similarly, residual platelets and varying freeze–thaw cycling can influence cfmiR profiles and may account for some of the discrepancies between the different blood fractions [108]. Although urine may not necessarily have as many cellular contaminants as blood, substantial differences in concentration, pH, and analyte (whole urine, supernatant, or sediment) have been documented to contribute to differences in EV quantity and have important implications in the validity of various urine studies [109]. Methodological standardization may become of special concern in future studies as newer technologies are developed to analyze liquid biopsies. Advances such as NGS, ddPCR, enhanced qPCR, biosensor-based platforms, and EV-focused assays may improve sensitivity, specificity, and multiplexing, but further contribute to already high heterogeneity in the field. Therefore, future progress and eventual clinical adoption depend not only on the advent of powerful technologies but also on careful harmonization of analytical workflows to ensure reproducible findings.

Widespread clinical adoption of their findings will also require further validation in multi-center cohorts with diverse populations, to produce robust evidence of cfmiR utility. In addition to important evidentiary considerations, high assay costs present an additional barrier to clinical adoption. Although qRT-PCR may be comparable in price to laboratory tests, ddPCR and NGS-based approaches remain comparatively more resource intensive and costly [107]. Further studies must, therefore, compare the cost-effectiveness of cfmiR approaches against current RCC detection and surveillance paradigms. Taken together, the future of the field may lie in moving from methodologically diverse, isolated biomarker discovery toward standardized and integrated models in which imaging and cfmiRs first stratify lesions, molecular classification informs biological context, and cfmiR and proteomic assays provide scalable support for clinical decision making in the diagnosis, prognosis, and monitoring of RCC.

## 4. Conclusions

cfmiR-based assays in urine and blood show some potential as noninvasive diagnostic tools for RCC, but evidence is largely immature. Urine cfmiR tests have achieved high specificity in initial, single-cohort studies, whereas blood-based (plasma/serum) tests and especially panels offer sensitivity for detecting RCC, including early stage tumors. Each fluid has possible advantages: urine provides a more local cfmiR signature with minimal invasiveness, while plasma/serum samples reflect systemic tumor signals and are more accessible. However, all of these cfmiR biomarkers and novel technologies remain investigational and current evidence lacks widespread, multi-cohort validation. Their clinical utility will depend on this validation and further standardization of methods. In the future, integrated approaches may significantly improve early RCC detection, as well as the identification of aggressive RCC tumors, offering a complement to current diagnostic and prognostic tools.

## Figures and Tables

**Table 1:** Summary of the cfmiRs found in serum samples from patients diagnosed with renal cell carcinoma.

Sample Type	Methodology (Assay/Vendor)	cfmiR Signature		Patients (*N*)	Control Group (*N*)	Performance for RCC vs. HD AUC (SE; SP)	Clinical Variable	Author (Year)	PMID
Serum	qRT-PCR (Bulge-Loop, RiboBio)	Single Panel	↑ miR-142-3p ↑ miR-223-3p * ↓ miR-30c-5p ↓ miR-200c-5p * ↓ miR-206 [miR-30c-5p, miR-142-3p, miR-206]	RCC (80)	HDs (84)	0.716 (66.25; 76.19) 0.685 (62.50; 78.57) * 0.769 (67.50; 83.33) 0.639 (78.75; 46.43) * 0.685 (75.00; 65.48) 3-miRNA panel 0.872 (81.25; 86.90)	Diagnosis	Lin S et al. (2025) [7]	40413252
Serum	qRT-PCR (Bulge-loop, HaiGene)	Single Panel	↑ miR-146b-5p ↓ miR-1-3p ↓ miR-129-5p ↓ miR-187-3p * ↓ miR-200a-3p * [miR-1-3p, miR-129-5p, miR-146b-5p]	ccRCC (112)	HDs (112)	0.681 (NA; NA) 0.642 (NA; NA) 0.618 (NA; NA) 0.712 (NA; NA) * 0.612 (NA; NA) * 3-miRNA panel 0.895 (89.30; 76.19)	Prognosis (OS); Diagnosis	Wen Z et al. (2024) [8]	38191389
Serum	qRT-PCR (TaqMan)	Single Panel	↑ miR-27a-3p ↑ miR-221-3p * ↓ miR-154-5p * ↓ miR-429 ↓ miR-10a-5p (n.s) miR-101-3p * [miR-27a-3p, miR-429, miR-10a-5p]	ccRCC (108)	HDs (112)	0.751 (56.25; 84.52) 0.690 (76.25; 59.52) * 0.618 (55.00; 64.29) * 0.745 (83.75; 53.57) 0.730 (71.25; 71.43) 0.678 (55.00; 76.19) * 3-miRNA panel 0.897 (85.00; 83.33)	Prognosis (OS); Diagnosis	Li X et al. (2024) [9]	37574436
Serum (EV isolated)	qRT-PCR (TaqMan)	Single	↑ miR-320d	Advanced ccRCC (100)	Localized ccRCC (146)	NA	Prognosis	Xue Y et al. (2023) [10]	37380801
Serum	qRT-PCR (Bulge-Loop, RiboBio)	Single Panel	↑ miR-155-5p ↑ miR-224-5p ↓ miR-1-3p ↓ miR-124-3p * ↓ miR-129-5p * ↓ miR-200b-3p [miR-1-3p, miR-155-5p, miR-200b-3p, miR-224-5p]	RCC (112)	HDs (112)	0.647 (64.63; 67.09) 0.730 (69.51; 70.73) 0.647 (87.80; 45.12) 0.633 (82.93; 45.12) * 0.743 (71.95; 70.73) * 0.747 (87.80; 52.44) 4-miRNA panel 0.903 (75.61; 93.67)	Prognosis (OS); Diagnosis	Li R et al. (2023) [11]	36727070
Serum	qRT-PCR (Bulge-Loop, RiboBio)	Single Panel	↑ miR-18a-5p ↑ miR-181b-5p **↓ miR-138-5p** ↓ miR-141-3p ↓ miR-200a-3p * ↓ miR-363-3p * [miR-18a-5p, miR-138-5p, miR-141-3p, miR-181b-5p]	RCC (108)	HDs (112)	0.704 (75.64; 64.63) 0.620 (73.08; 50.62) 0.771 (64.10; 84.15) 0.743 (92.31; 53.66) 0.701 (62.82; 68.29) * 0.646 (56.41; 69.51) * 4-miRNA panel 0.908 (80.77; 88.89)	Prognosis (OS); Diagnosis	Li R et al. (2022) [12]	35938021
Serum	qRT-PCR (PrimeScript, TaKaRa)	Single Panel	↑ miR-150–5p ↑ miR-21–5p **↓ miR-145–5p** ↓ miR-146a-5p ↓ miR-17-5p * ↓ miR-20a-5p * [miR-145–5p, miR-146a-5p, miR-150–5p, miR-21–5p]	RCC (123)	HDs (118)	0.717 (NA; NA) 0.691 (NA; NA) 0.827 (NA; NA) 0.785 (NA; NA) 0.629 (NA; NA) * 0.704 (NA; NA) * 4-miRNA panel 0.938 (90.79; 93.75)	Diagnosis	Chen X et al. (2021) [13]	34628264
Serum (EV isolated)	qRT-PCR (TaqMan)	Single	↑ miR-4525	Localized ccRCC (9); Advanced ccRCC (9)	HDs (7)	NA (NA; NA)	Diagnosis	Muramatsu-Maekawa Y et al. (2021) [14]	33893078
Serum	qRT-PCR (PrimeScript, TaKaRa)	Single Panel	↑ miR-196a-5p ↓ miR-20b-5p **↓ miR-30a-5p** [miR-20b-5p, miR-30a-5p, miR-196a-5p]	RCC (110)	HDs (110)	0.717 (NA; NA) 0.780 (NA; NA) 0.787 (NA; NA) 3-miRNA panel 0.938 (92.50, 80.80)	Diagnosis	Huang G et al. (2020) [15]	32823234
Serum	qRT-PCR (Exiqon)	Single Panel	↑ miR-21-5p **↓ miR-210-3p** [miR-210-3p; miR-21-5p]	pRCC type 1 (34) pRCC type 2 (33)	HDs (33)	0.574 (NA; NA) 0.708 (NA; NA) 2-miRNA panel 0.718 (NA; NA);	Diagnosis	Kalogirou C et al. (2020) [16]	32676415
Serum	qRT-PCR (PrimeScript, TaKaRa)	Single Panel	↑ miR-224-5p **↓ miR-34b-3p** ↓ miR-182-5p ↓ miR-129-2-3p * (n.s.) miR-149-5p * [miR-224-5p, miR-34b-3p, miR-182-5p]	RCC (146)	HDs (150)	0.692 (NA; NA) 0.778 (NA; NA) 0.745 (NA;NA) 0.687 (NA; NA) * 0.545 (NA; NA) * 3-miRNA panel 0.879 (80.0, 69.0)	Diagnosis	Huang G et al. (2020) [17]	32556891
Serum	qRT-PCR (Hairpin-it, GenePharma)	Single Panel	**↑ miR-885-5p** ↓ miR-508-3p [miR-508-3p, miR-885-5p]	ccRCC (85)	HDs (35)	0.87 (NA; NA) 0.80 (NA; NA) 2-miRNA panel 0.90 (NA; NA)	Diagnosis	Liu S et al. (2019) [18]	31661117
Serum	qRT-PCR (Invitrogen)	Single	↑ miR-210 **↑ miR-210 (Exosomal)**	ccRCC (45)	HDs (30)	0.7888 (67.5; 70.0) 0.8779 (82.5; 80.0)	Prognosis (Fuhrman Grade); Diagnosis	Wang X et al. (2019) [19]	30304555
Serum	qRT-PCR (miScript, Qiagen)	Single Panel	↓ miR-122-5p **↓ miR-206** [miR-122-5p, miR-206]	ccRCC (68) BRT (47)	HDs (28)	0.705 (NA; NA) 0.733 (57.1; 83.8) 2-miRNA panel 0.733 (NA; NA)	Prognosis (OS, CSS, PFS); Diagnosis	Heinemann F et al. (2018) [20]	29410711
Serum	qRT-PCR (Sangon Biotech)	Single	↑ miR-429	RCC (27)	HDs (28)	NA (NA; NA)	Prognosis (Recurrence)	Wang Z et al. (2017) [21]	29332334
Serum (EV isolated)	qRT-PCR (miScript, Qiagen)	Single	↑ miR-210 **↑ miR-1233**	ccRCC (82)	HDs (80)	0.69 (70; 62.2) 0.82 (81; 76)	Diagnosis	Zhang W et al. (2018) [22]	28753793
Serum	qRT-PCR (RT Kit, Thermo Scientific)	Single Panel	**↑ miR-21** ↑ miR-106a [miR-21, miR-106a]	ccRCC (30)	HDs (30)	0.865 (77.3; 96.4) 0.819 (86.7; 70.0) 2-miRNA panel NA (86.7; 66.7)	Diagnosis	Tusong H et al. (2017) [23]	27814278
Serum	qRT-PCR (miScript, Qiagen)	Single Panel	**↑ miR-1233** ↓ miR-34a ↓ miR-141 [miR-141, miR-1233] [miR-1233; miR-34a] [miR-141; miR-34a]	ccRCC (30)	HDs (15)	0.97 (93.33; 100) 0.92 (80.7; 80.00) 0.78 (75; 73.33) 2-miRNA panel NA (100.0; 73.33) 2-miRNA panel NA (96.6; 80.0) 2-miRNA panel NA (73.3; 60.0)	Diagnosis	Yadav S et al. (2017) [24]	28336290
Serum	qRT-PCR (Promega)	Single	↑ miR-210	RCC (32)	HDs (32)	NA (NA; NA)	Diagnosis	Liu T et al. (2016) [25]	26985942
Serum	qRT-PCR (TaqMan)	Single Panel	**↑ miR-378a-5p** ↑ miR-210 [miR-378a-5p, miR-210]	RCC (195)	HDs (100)	0.82 (NA; NA) 0.74 (NA; NA) 2-miRNA panel 0.848 (80; 78)	Prognosis (DFS); Diagnosis	Fedorko M et al. (2015) [26]	26426010
Serum	qRT-PCR (TaqMan)	Panel	↑ miR-193a-3p ↑ miR-362 ↓ miR-572 ↓ miR-28-5p ↓ miR-378a-5p [miR-193a-3p, miR-362, miR-572, miR-28-5p, miR-378]	RCC (107)	HDs (107)	5-miRNA panel 0.807 (80; 71)	Diagnosis	Wang C et al. (2015) [27]	25556603
Serum	qRT-PCR (TaqMan)	Single	↑ miR-210	ccRCC (34)	HDs (23)	0.77 (65; 83)	Diagnosis	Iwamoto H et al. (2014) [28]	24212760
Serum	qRT-PCR (miScript, Qiagen)	Single	↑ miR-210	ccRCC (68)	HDs (42)	0.874 (81.0; 79.4)	Diagnosis	Zhao A et al. (2013) [29]	23064048
Serum	qRT-PCR (TaqMan)	Single	↑ miR-378 #	RCC (117)	HDs (123)	0.728 (NA; NA) (discovery cohort only)	Diagnosis	Hauser S et al. (2012) [30]	22542158
Serum	qRT-PCR (TaqMan)	Single Panel	↑ miR-378a-5p **↓ miR-451** [miR-378a-5p, miR-451]	RCC (90)	HDs (35)	0.71 (70; 60) 0.77 (81; 77) 2-miRNA panel 0.86 (81; 83)	Diagnosis	Redova M et al. (2012) [31]	22440013
Serum	qRT-PCR (TaqMan)	Single	↑ miR-1233	RCC (84)	HDs (93)	0.588 (77.4; 37.6)	Diagnosis	Wulfken L et al. (2011) [32]	21984948

Abbreviations: AUC, area under the curve; cfmiR, cell-free microRNA; ccRCC, clear cell renal cell carcinoma; pRCC, papillary renal cell carcinoma; HD, healthy donor; RCC, renal cell carcinoma; qRT-PCR, quantitative real-time polymerase chain reaction; miR, microRNA; NA, not assigned; n.s., not significant; SE, sensitivity; SP, specificity. Up and down arrows indicate the reported relative increase or decrease in that cfmiR in RCC patients versus HDs. Asterisks indicate miRNAs that were significantly different between groups and characterized with either AUC, SE, and/or SP but excluded from a diagnostic panel due to poor performance. If a study evaluated more than one cfmiR, the signature with the highest AUC is bolded. Sensitivity and specificity values reflect performance in distinguishing RCC (all or specific subtype) from healthy controls (and in some cases from benign renal masses as noted). # Found to be not significantly different between RCC/HDs in validation cohort of 117 RCC and 123 HDs.

**Table 2:** Summary of the cfmiRs found in plasma samples from patients diagnosed with renal cell carcinoma.

Sample Type	Methodology (Assay/Vendor)		cfmiR Signature	Patients (*N*)	Control Group (*N*)	Performance for RCC vs. HDs AUC (SE; SP)	Clinical Variable	Author (Year)	PMID
Plasma	NGS (HTG WTA assay)	Single	↑ miR-1275	RCC (18)	HDs (73)	0.801 (72.22; 63.01)	Diagnosis	Bustos M et al. (2024) [33]	37791385
Plasma	ddPCR (TaqMan)	Single Panel	↑ miR-182-5p **↑ miR-375-3p** [miR-182-5p, miR-375-3p]; ↑ miR-155-5p ↑ miR-182-5p * ↓ miR-375-3p [miR-155-5p, miR-375-3p]	RCC (119); PCa (76); BlCa (73);	HDs (74)	0.615 (36.94; 90.54) 0.711 (36.94; 90.54) 2-miRNA panel NA (64.93; 81.08) 0.581 (58.82; 61.74) 0.656 (67.23; 67.11) 0.596 (78.99; 40.94) 2-miRNA panel NA (47.90; 80.54)	Diagnosis	Sequeira JP et al. (2023) [34]	37762193
Plasma	ddPCR (TaqMan)	Panel	**↑ miR-155-5p** ↓ miR-21-5p [miR-21-5p, miR-155-5p]	ccRCC (87) pRCC (15) chRCC (22)	HDs (64); Oncocytomas (15)	0.669 (62.90; 64.06) 0.610 (39.05; 90.63) 2-miRNA panel NA (82.66; 51.13)	Diagnosis	Sequeira JP et al. (2022) [35]	35205607
Plasma	qRT-PCR (TaqMan)	Single Panel	↓ miR-150 **↓ miR-451** [miR-451, miR-26b#]	ccRCC (94)	HDs (100)	NA (NA; NA) 0.64 (NA; NA) 2-miRNA panel 0.80 (NA; NA)	Prognosis (OS, PFS); Diagnosis	Chanudet E et al. (2017) [36]	28639257

Abbreviations: AUC, area under the curve; cfmiR, cell-free microRNA; ccRCC, clear cell renal cell carcinoma; pRCC, papillary renal cell carcinoma; chRCC, chromophobe renal cell carcinoma; HD, healthy donor; RCC, renal cell carcinoma; qRT-PCR, quantitative real-time polymerase chain reaction; ddPCR, digital droplet polymerase chain reaction; miR, microRNA; NA, not assigned; SE, sensitivity; SP, specificity. Up and down arrows indicate the reported relative increase or decrease in that cfmiR in RCC patients versus HDs. Asterisks indicate miRNAs that were significantly different between groups and characterized with either AUC, SE, and/or SP but excluded from a diagnostic panel due to poor performance. If a study evaluated more than one cfmiR, the signature with the highest AUC is bolded. Sensitivity and specificity values reflect performance in distinguishing RCC (all or specific subtype) from healthy controls (and in some cases, from benign renal masses as noted).

**Table 3:** Summary of the cfmiRs found in urine samples from patients diagnosed with RCC.

Sample Type	Methodology (Assay/Vendor)	cfmiR Signature		Patients (*N*)	Control Group (*N*)	Performance for RCC vs. HD AUC (SE; SP)	Clinical Variable	Author (Year)	PMID
Urine (Supernatant)	qRT-PCR (TaqMan)	Single	↑ miR-191-5p ↑ miR-324-3p ↑ miR-93-5p ↑ miR-30b-5p ↑ miR-186-5p	ccRCC (117)	ccRCC (117) (post-nephrectomy)	NA (NA; NA)	Prognosis	Borges Dos Reis R et al. (2024) [6]	37945405
Whole Urine	NGS (HTG WTA assay)	Single	↑ miR-1275	RCC (17)	HDs (16)	0.933 (92; 93.75)	Diagnosis	Bustos M et al. (2024) [33]	37791385
Whole Urine	qRT-PCR (miScript, II, Qiagen)	Single Panel	**↑ miR-122-5p** ↑ miR-1271-5p ↑ miR-15b-5p [miR-122-5p, miR-1271-5p, miR-15b-5p]	RCC (28)	HDs (28)	0.80 (NA; NA) 0.66 (NA; NA) 0.65 (NA; NA) 3-miRNA panel 0.81 (96; 65)	Diagnosis	Cochetti G et al. (2022) [37]	35267420
Urine (Supernatant)	qRT-PCR (Exiqon)	Panel	↑ miR-200a-3p ↑ miR-34a-5p ↑ miR-365a-3p ↑ let-7d-5p ↑ miR-205-5p [miR-200a-3p, miR-34a-5p, miR-365a-3p]	ccRCC (29) pRCC (16) chRCC (6)	HDs (32) Angiomyolipoma (13)	0.65 (NA; NA)	Diagnosis	Oto J et al. (2021) [38]	34360679
Whole Urine	qRT-PCR (miScript, Qiagen)	Panel	**↑ miR-122** ↑ miR-1271 ↑ miR-15b [miR-122, miR-1271, miR-15b]	RCC (13)	HDs (14)	0.82 (NA; NA) 0.79 (NA; NA) 0.59 (NA; NA) 3-miRNA panel 0.96 (100; 86)	Diagnosis	Cochetti G et al. (2020) [39]	33277569
Urine (Precipitate)	qRT-PCR (TaqMan)	Single	↑ miR-30a-5p^me^	Cohort 1: ccRCC (53) Cohort 2: RCC (171)	Cohort 1: HDs (57) Cohort 2: HDs (85)	0.670 (63; 67)	Prognosis (MFS, DFS, DSS) Diagnosis	Outeiro-Pinho G et al. (2020) [40]	32487203
Whole Urine	qRT-PCR (miScript II, Qiagen)	Single	↑ miR-210-3p	RCC (21)	HDs (16)	NA (NA; NA)	Prognosis (Metastasis)	Petrozza V et al. (2021) [41]	31771042
Urine (EV isolated)	qRT-PCR (Life Technologies)	Single	↓ miR-30c-5p	ccRCC (70)	HDs (30)	0.8192 (68.57; 100)	Diagnosis	Song S et al. (2019) [42]	31342628
Whole Urine	qRT-PCR (TaqMan)	Single	↑ miR-15a	ccRCC (22) pRCC (16) chRCC (14)	HDs (14) Oncocytoma (8) Papillary adenoma (2) Angiomyolipoma (4)	0.955 (100, 98.1)	Diagnosis	Mytsyk Y et al. (2018) [43]	29549624
Whole Urine	qRT-PCR (miScript II, Qiagen)	Single	↑ miR-210-3p	RCC (38)	HDs (10)	NA (NA; NA)	Diagnosis	Petrozza V et al. (2017) [44]	29050224
Urine (Supernatant)	qRT-PCR (TaqMan)	Single Panel	**↑ let-7a** ↑ let-7b ↑ let-7c ↑ let-7d ↑ let-7e ↑ let-7g [let-7a, let-7b, let-7c, let-7d, let-7e, let-7g]	RCC (69)	HDs (36)	0.8307 (71; 81) 0.75 (73; 67) 0.67 (65; 62) 0.66 (66; 61) 0.65 (62; 61) 0.69 (70; 60) 6 miRNA panel 0.83 (NA; NA)	Diagnosis	Fedoroko et al. (2017) [45]	28694731
Urine (Supernatant)	qRT-PCR (miScript, Qiagen)	Single	↑ miR-210	RCC (75)	HDs (45)	0.76 (57.8, 80)	Diagnosis	Li G et al. (2017) [46]	28089386

Abbreviations: AUC, area under the curve; cfmiR, cell-free microRNA; ccRCC, clear cell renal cell carcinoma; pRCC, papillary renal cell carcinoma; chRCC, chromophobe renal cell carcinoma; HD, healthy donor; RCC, renal cell carcinoma; qRT-PCR, quantitative real-time polymerase chain reaction; miR, microRNA; NA, not assigned; SE, sensitivity; SP, specificity. Up and down arrows indicate the reported relative increase or decrease in that cfmiR in RCC patients versus HDs. If a study evaluated more than one cfmiR, the signature with the highest AUC is bolded. Sensitivity and specificity values reflect performance in distinguishing RCC (all or specific subtype) from healthy controls (and in some cases from benign renal masses as noted).

**Table 4:** Summary of shared cfmiRs and their associated expression levels between different kidney diseases and RCC.

cfmiR	Disease	cfmiR Levels in Serum, Plasma, or Urine	Author (Year)
miR-223	CKD	Decreased Decreased Decreased Decreased	Carmona A et al. (2020) [76] Fourdinier O et al. (2019) [77] Fujii R et al. (2019) [78] Ulbing M et al. (2017) [79]
	RCC	Increased	Lin S et al. (2025) [7]
miR-155	CKD	Decreased Decreased Increased * Increased	Chen N.X. et al. (2013) [80] Donderski R et al. (2021) [81] Donderski R et al. (2021) [81] Eckersten D et al. (2017) [82]
	DKD	Decreased Decreased * Increased * Decreased †	Akhbari M et al. (2019) [83] Barutta F et al. (2013) [84] Wang J et al. (2019) [85] Wang J. et al. (2019) [85]
	RCC	Increased Increased † Increased †	Li R et al. (2022) [12] Sequeira JP et al. (2023) [34] Sequeira JP et al. (2022) [35]
miR-21	CKD	Increased * Decreased Increased *	Donderski R et al. (2021) [81] Donderski R et al. (2021) [81] Lange T et al. (2019) [86]
	DKD	Increased † Increased † Increased * Increased Increased * Increased * Increased	Florijn B.W. et al. (2019) [87] Fouad M et al. (2020) [88] Milas O et al. (2018) [89] Petrica L et al. (2019) [90] Petrica L et al. (2019) [90] Zang J et al. (2019) [91] He F et al. (2014) [92]
	RCC	Increased Increased Increased Decreased †	Chen X et al. (2021) [13] Kalogirou C et al. (2020) [16] Tusong H et al. (2017) [23] Sequeira JP et al. (2022) [35]
miR-15a	DKD	Increased	He F et al. (2014) [92]
	RCC	Increased *	Mytsyk Y et al. (2018) [43]
miR-124	DKD	Increased * Decreased Increased *	Milas O et al. (2018) [89] Petrica L et al. (2019) [90] Petrica L et al. (2019) [90]
	RCC	Decreased	Li R et al. (2023) [11]
let-7a	DKD	Decreased † Decreased * Decreased †	Park S et al. (2017) [93] Park S et al. (2017) [93] Wang J et al. (2019) [85]
	RCC	Increased	Fedorko et al. (2017) [45]
miR-30b	DKD	Increased Decreased † Decreased * Decreased *	He F et al. (2014) [92] He F et al. (2014) [92] Park S et al. (2022) [93] Zang J et al. (2019) [91]
	RCC	Increased *	Borges Dos Reis R et al. (2024) [6]

Abbreviations: CKD, chronic kidney disease; DKD, diabetic kidney disease. Asterisks (*) indicate urine findings, obelisks (†) indicate plasma findings, and no symbol follows serum findings. cfmiR information in CKD and DKD was extracted from the Motshwari et al. (2023) review and citations can be found there [75].

## Data Availability

All data supporting the conclusions of this article are publicly available.

## Abbreviations

AUC	area under the curve
BlCa	bladder cancer
BRT	benign renal tumor
ccRCC	clear cell renal cell carcinoma
chRCC	chromophobe renal cell carcinoma
cfDNA	cell-free DNA
cfmiRs	cell-free microRNAs
cfNA	cell-free nucleic acid
CI	confidence interval
CKD	chronic kidney disease
CT	computed tomography
CSS	cancer-specific survival
CTCs	circulating tumor cells
ddPCR	droplet digital polymerase chain reaction
DFS	disease-free survival
DKD	diabetic kidney disease
EVs	extracellular vesicles
FH	fumarate hydratase
HD	healthy donor
HR	hazard ratio
ICI	immune checkpoint inhibitor
MFS	metastasis-free survival
miRNA	microRNA
mRNA	messenger RNA
MRI	magnetic resonance imaging
NGS	next-generation sequencing
NR	not reported
OS	overall survival
PCa	prostate cancer
PCR	polymerase chain reaction
PD-L1	programmed cell death ligand-1
PD-1	programmed cell death protein 1
PFS	progression-free survival
PMID	PubMed Identifier
pRCC	papillary renal cell carcinoma
qRT-PCR	quantitative reverse transcription polymerase chain reaction
RCC	renal cell carcinoma
ROC	receiver operating characteristic
SE	sensitivity
SP	specificity
TME	tumor microenvironment
